# Effect of Hypoxic Exercise with Different Oxygen Concentrations on Metabolic Outcomes in Obesity: A Systematic Review and Network Meta-Analysis

**DOI:** 10.3390/life16020231

**Published:** 2026-02-01

**Authors:** Kai Gao, Shuting Liu, Chengyu Zhou, Diandong Lang, Huaichuan Zhang

**Affiliations:** 1 Strength and Conditioning Training College, Beijing Sports University, Beijing 100084, China; tylp7658@163.com (K.G.); chengyuzhou0102@gmail.com (C.Z.);; 2 College of Sports and Health, Linyi University, Linyi 276005, China; 3 College of Education, Beijing Sports University, Beijing 100084, China

**Keywords:** oxygen concentration, obesity, glucose metabolism, lipid metabolism, hypoxic exercise, network meta-analysis

## Abstract

Objective: This study aimed to systematically evaluate the effects of hypoxic exercise at different oxygen concentrations on body composition, glucose metabolism, and lipid metabolism in individuals with obesity, and to explore potential optimal oxygen concentration ranges to inform personalized hypoxic exercise prescriptions. Methods: We searched databases including the Cochrane Library, PubMed, Web of Science, Embase, and CNKI for randomized controlled trials and pre-post studies on hypoxic exercise interventions in obese populations published before 30 June 2025. A network meta-analysis was performed using Stata 18.0 software to assess the effects of various oxygen concentrations on BMI, FBG, FINS, HOMA-IR, TG, LDL-C, and HDL-C. Subgroup analyses were conducted to explore sources of heterogeneity. Results: Fourteen studies with a total sample size of 189 participants were included. The meta-analysis revealed that exercise in a moderate hypoxic environment (12–14% FiO_2_) significantly reduced BMI, FBG, TG, and LDL-C. Notably, hypoxic exercise did not show a statistically significant effect on HDL-C. In contrast, a higher oxygen concentration (≥15% FiO_2_) was more effective for improving FINS and HOMA-IR. Conclusion: Hypoxic exercise can significantly improve the body composition, glucose metabolism and lipid metabolism indicators of obese people. Tailored exercise in specific hypoxic environments provides preliminary evidence for a non-pharmacological intervention strategy in obesity management.

## 1. Introduction

The global incidence of obesity and related metabolic diseases has risen steadily, establishing itself as a major and pressing public health challenge. Obesity significantly elevates the risk of nusmerous chronic conditions, including cardiovascular disease, metabolic syndrome, diabetes, hypertension, and dyslipidemia, and is also strongly associated with the incidence of various cancers [1,2]. According to global estimates, over one billion people (≈13% of the world’s population) live with obesity [3]. In China, 402 million adults (≥25 years) were overweight or obese in 2021 [4,5]. Projected to reach 627 million by 2050, with 35.2 million obese children and adolescents expected, posing a severe public health threat [6]. Exercise intervention, a key non-pharmacological approach for managing obesity and metabolic disorders, is widely used in clinical and health management practice [7,8]. However, the efficacy of traditional normoxic exercise may be limited by poor compliance, insufficient metabolic adaptation, and lengthy intervention cycles in some populations [9]. Notably, individual responses to normoxic exercise vary due to differences in intensity, duration, and compliance, leading to inconsistent therapeutic effects [10]. Some individuals with obesity may not achieve the desired therapeutic effect through normoxic exercise due to insufficient metabolic adaptation or poor exercise tolerance. Hypoxic training may address this limitation by inducing beneficial metabolic remodeling [11].

Hypoxic exercise developed from the concept of high-altitude training, has emerged as a prominent topic in metabolic intervention research due to its unique advantages in remodeling energy metabolism [12]. It is defined as an exercise modality conducted under conditions of environmental hypoxia, where decreased oxygen supply to tissues reduces arterial oxygen saturation [13]. Evidence suggests that hypoxic exercise is more effective than normoxic exercise in improving body composition, glucose metabolism, and dyslipidemia in individuals with obesity, making it particularly suitable for those who experience limited benefits from or have difficulty adhering to traditional exercise regimens [14,15,16]. This effect is enhanced when aerobic exercise is combined with resistance training, leading to more significant reductions in body fat percentage [17]. Appropriately dosed intermittent hypoxia can confer metabolic advantages and reduce obesity risk while avoiding the adverse health effects associated with excessive or frequent hypoxia exposure. In some studies, moderate-altitude hypoxia is reported to be more effective for obesity management than high-altitude or extreme-altitude exposure [18,19]. Furthermore, hypoxic exercise has been shown to improve inflammatory biomarkers in obese elderly women, indicating potential value for cardiovascular disease prevention [20]. In terms of metabolic regulation, hypoxic exercise effectively promotes blood glucose homeostasis by enhancing peripheral glucose uptake and insulin sensitivity, with a more pronounced effect than exercise under normoxic conditions. The underlying mechanisms primarily involve the enhancement of fat oxidation and glucose utilization, increased energy expenditure, and the promotion of erythropoiesis and angiogenesis via metabolic pathways such as AMPK, collectively improving metabolic function [21,22,23]. However, the efficacy of hypoxic exercise depends on key factors such as oxygen concentration, exercise mode, and duration. Poorly designed interventions may increase cardiovascular risk and other metabolic burdens.

While research on hypoxic exercise is growing, findings regarding its efficacy under different levels of hypoxia vary considerably, and a systematic, quantitative synthesis is lacking. This is particularly evident in the selection of the FiO_2_, where the absence of a scientific consensus leads to inconsistent results in practice and a lack of specific, actionable guidance. Therefore, this study will employ a network meta-analysis to comprehensively evaluate the effects of varying FiO_2_ levels during hypoxic exercise on body composition, glucose metabolism, and lipid metabolism in individuals with obesity. The aims are to clarify the optimal FiO_2_ range, refine intervention strategies, and advance the development of a personalized, precise hypoxic exercise prescription system. This work seeks to provide a scientific foundation and practical guidance for non-pharmacological interventions targeting obesity and related metabolic disorders.

## 2. Materials and Methods

### 2.1. Search Strategy

Based on the systematic literature review and meta-analysis methods recommended in the PRISMA guidelines for the standard process of meta-analysis proposed by Moher et al. [24], literature search and screening were conducted. The research question was a meta-analysis of the effects of hypoxic exercise on glucose metabolism and lipid metabolism indicators in obese people. The databases retrieved in this study included Cochrane Library, PubMed, Web of Science, Embase and CNKI. The specific Boolean search strategy was revised to: (hypoxic OR hypoxia) AND (exercise OR training) AND (obesity OR overweight) AND (glucose metabolism OR lipid metabolism OR randomized controlled trial OR RCT). If the data in the original publication is missing or unclear, an attempt will be made to contact the corresponding author via email. This study is based on the PRISMA (preferred reporting items for systematic reviews and meta-analysis) statement and has been registered on the PROSPERO website. The registration number is CRD 420251166378.

### 2.2. Selection Process

Manual deduplication of retrieved records was performed by independent reviewers using EndNote X9. Following deduplication, the records were exported and allocated to two independent researchers for title and abstract screening against the predefined eligibility criteria. Disagreements were resolved through discussion between the two reviewers, with adjudication by a third researcher if consensus could not be reached. The same two reviewers then independently assessed the full texts of potentially eligible articles. Any disagreements at this stage were resolved using the same consensus and adjudication protocol.

### 2.3. Eligibility Criteria

The inclusion and exclusion criteria were developed according to the Population, Intervention, Comparison, Outcome, Study design (PICOS) framework, as detailed below:

The inclusion criterion was population (P): Participants were adults classified as overweight or obese. This classification was based on the Chinese Guidelines for Prevention and Control of Overweight and Obesity in Adults, which define the conditions as follows, obesity is defined as a BMI > 28 kg/m^2^, and overweight is defined as a BMI > 24 kg/m^2^. Intervention (I): A structured exercise program, defined as a standardized exercise scheme including clear exercise type, intensity, frequency, single session duration, and the normoxia control group must be included. Comparison (C): Eligible comparators included non-exercise control conditions, such as wait-list controls, placebo, or usual care (e.g., nutritional advice or general lifestyle counseling without a structured exercise component). Outcomes (O): Studies were required to report data for at least one of the following primary outcomes, measured both before and after the intervention: BMI, glucose metabolism, or lipid metabolism. Study Design (S): Only RCTs that included quantitative pre- and post-intervention assessments with between-group comparisons were eligible.

The exclusion criteria were as follows: (1) Insufficient data or failure to report the specific oxygen concentration. (2) Animal studies. (3) Interventions that do not include a structured exercise component or involve concurrent primary interventions such as pharmacological therapy, nutritional supplementation, or surgery. (4) Participants diagnosed with major metabolic comorbidities (e.g., diabetes, hypertension, non-alcoholic fatty liver disease) or those using weight-loss medication. (5) Studies with outcomes irrelevant to the metabolic indicators of interest or from which data cannot be extracted.

### 2.4. Data Extraction

Data extraction was performed independently by the two reviewers who conducted the screening, using a standardized extraction form. From each included study, both reviewers extracted the following details: authorship and publication year, study design, participant demographics, intervention characteristics, and outcome data. A third reviewer cross-checked all extracted entries for accuracy. Any discrepancies were resolved through consensus discussion between the original reviewers, with a fourth researcher available for adjudication if needed. When data were missing or reported only in figures, the corresponding authors were contacted to obtain the necessary information. Studies from which essential data could not be retrieved through this correspondence were excluded from the final quantitative synthesis.

This study extracted the mean, standard deviation, and sample size reported for each group before and after the intervention. Effect sizes were pooled by calculating the mean change (difference between post-intervention and pre-intervention values) and its standard deviation for each outcome. The mean change for each intervention group was computed using the following Formula (1):

(1) $${MD}_{diff}=M_{post}-M_{pre}$$

where ${MD}_{diff}\;$ represents the raw mean difference, ${MD}_{diff}$ represents the reported mean postintervention, and $M_{pre}$ represents the reported mean preintervention.

If the study reported only confidence intervals, they were converted to SDs via the following Formula (2):

(2) $$SD=\sqrt{N}(\frac{{CI}_{high}-{CI}_{low}}{2t})$$

where *SD* is the standard deviation, *N* is the group sample size, ${CI}_{high}$ is the upper limit of the confidence interval, ${CI}_{low}$ is the lower limit of the confidence interval, and *t* is the *t* distribution with *N* − 1 degrees of freedom at the respective confidence level.

The *SD* of the difference in means (${SD}_{diff}$) was calculated via the following Formula (3):

(3) $${SD}_{diff}=\sqrt{{{SD}_{pre}}^{2}+{{SD}_{post}}^{2}-2r\times {SD}_{pre}\times {SD}_{post}}$$

where ${SD}_{diff}$ is the standard deviation of the difference in means, ${SD}_{pre}$ is the standard deviation from preintervention, and ${SD}_{post}$ is the standard deviation from postintervention. As the original studies included in the meta-analysis did not report Pearson’s correlation coefficients (*r*) for pre- and postintervention outcomes, we used 0.5, which was a ${SD}_{change}$ based on the recommendations in the Cochrane Library. It was calculated via the Formula (4):

(4) $$r=\frac{{{SD}_{pre}}^{2}+{{SD}_{post}}^{2}-{{SD}_{change}}^{2}}{2\times {SD}_{pre}\times {SD}_{post}}$$

### 2.5. Risk of Bias and Quality of Methods Assessment

The Cochrane Collaboration’s Risk of Bias Tool was used to assess bias in six dimensions: random sequence generation, allocation concealment, blinding (participant/researcher blinding), data completeness, selective reporting, and other biases. Disagreements between reviewers were resolved through discussion whenever possible. If a consensus could not be reached, a third independent reviewer was consulted for adjudication.

Additionally, the Physiotherapy Evidence Database (PEDro) scale was used to assess the methodological quality of the included studies. The combination of both tools provides a more comprehensive evaluation of study quality, complementing each other’s strengths. This approach is consistent with previous systematic reviews on exercise interventions. The PEDro scale rates studies on a scale from 0 to 10, with higher scores indicating greater methodological rigor. Based on conventional interpretation of the scale, with scores of ≥6 (high quality), scores of 4–5 (moderate quality), and scores ≤ 3 (low quality).

### 2.6. Statistical Analysis

All statistical analyses were conducted using Review Manager 5.4 and Stata 18.0. As all outcome indicators were continuous variables, the MD with 95% (CI served as the effect measure for outcomes assessed with consistent methods and units. For outcomes measured with different instruments or scales, the SMD with 95% CI was employed. The methodological quality of the included RCTs was evaluated using the Cochrane Risk of Bias tool, covering six dimensions: random sequence generation, allocation concealment, blinding (participant/researcher blinding), data completeness, selective reporting, and other biases. The leave-one-out method was used to assess the robustness of the meta-analysis results. By sequentially excluding each included study and recalculating the combined effect size, the impact of a single study on the overall result was observed. If the direction and statistical significance of the combined effect size did not change significantly, the result was considered robust. Stata 18.0 software was used for league table analysis, network relationship analysis, and SUCRA ranking to compare the effects of different oxygen concentration groups on each outcome indicator.

### 2.7. Certainty of the Evidence

The certainty of evidence for each study was formally appraised using the GRADE framework. This assessment was performed independently by two researchers, who resolved any discrepancies through consensus discussion. The overall certainty of the synthesized evidence was then rated as high, moderate, low, or very low, directly informing the interpretation of the results. This comprehensive assessment rates evidence as follows: (1) the risk of bias, downgraded by one level if “some concerns” and two levels if “high risk” of bias; (2) inconsistency, downgraded by one level when the impact of statistical heterogeneity (I^2^) is moderate (>25%) and by two levels when high >75%; (3) imprecision, downgraded by one level when statistical power < 80% and if there was no clear direction of the effects; and (4) risk of publication bias, downgrade one level if Egger’s test < 0.05.

The certainty of evidence for each outcome was appraised using the GRADE framework. Two reviewers independently conducted the assessments. Any disagreements were resolved through consensus discussion. The overall certainty of the synthesized evidence for each outcome was then classified as high, moderate, low, or very low. Evidence was downgraded from an initial rating of “high” based on the following criteria: (1) Risk of bias: Downgraded by one level for “some concerns” and by two levels for a “high risk” of bias, as assessed by the Cochrane Risk of Bias Tool. (2) Inconsistency: Downgraded by one level for substantial unexplained heterogeneity (I^2^ > 50%) and by two levels for considerable heterogeneity (I^2^ > 75%). (3) Imprecision: Downgraded by one level if the 95% CI included both a significant benefit and no benefit (or harm), or if the optimal information size was not met. (4) Publication bias: Downgraded by one level if significant asymmetry was indicated by a funnel plot and corroborated by Egger’s test (*p* < 0.05).

## 3. Results

### 3.1. Studies Retrieved

**Figure 1:**
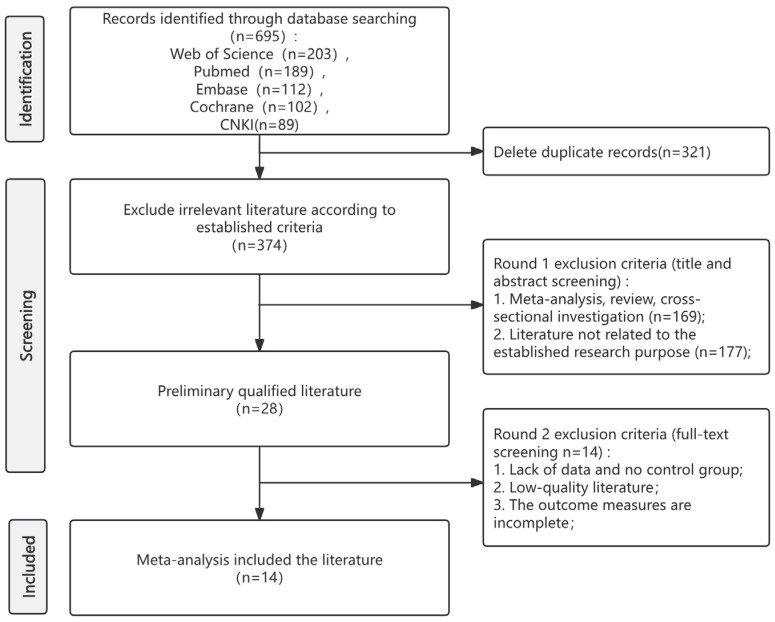
PRISMA flow diagram of study selection.

Systematic database search yielded 695 records. Following the removal of 321 duplicates, 374 records proceeded to title and abstract screening. This process identified 28 articles for full-text review, which were then assessed against the predefined eligibility criteria. Ultimately, 14 studies met all criteria for inclusion in the systematic review and meta-analysis. The complete study selection process is illustrated in the PRISMA flow diagram (Figure 1). The specific information of the PRISMA checklist can be found in Table S1.

### 3.2. Characteristics of the Included Studies

Following the screening process and full-text assessment, 14 studies [25,26,27,28,29,30,31,32,33,34,35,36,37,38] meeting the inclusion criteria were selected for analysis, encompassing a total sample size of 189 participants. All included studies utilized a pre-post design within the hypoxic intervention groups. The effect size for the meta-analysis was calculated as the mean change from baseline within these groups; data from normoxic control groups were not incorporated. In the included studies, the FiO_2_ ranged from 12% to 16.4%. There may be considerable heterogeneity in terms of duration and types. Intervention duration ranged from 3 to 32 weeks, and exercise modalities included AT, RT, HIIT, and their combinations [22,23,39]. The basic characteristics of the included studies are presented in Table 1.

In this study, FiO_2_ was categorized into 12–13%, 13–14%, 14–15%, and ≥15% based on two key considerations: (1) clinical relevance: previous hypoxic exercise studies [27,32,37] have consistently used these thresholds to distinguish mild (≥15%), moderate (12–14%), and severe (<12%) hypoxia, with moderate hypoxia (12–14%) widely regarded as a therapeutic window for metabolic improvement. (2) Data-driven grouping: the included studies (Table 1) showed that the values of inhaled oxygen concentration naturally clustered around these ranges, avoiding arbitrary divisions that might distort effect estimates. Due to limited data and potential safety risks, e.g., inducing stress responses [40], severe hypoxia (<12%) was not included.

### 3.3. Indicators Related to Body Composition

#### Body Mass Index

**Figure 2:**
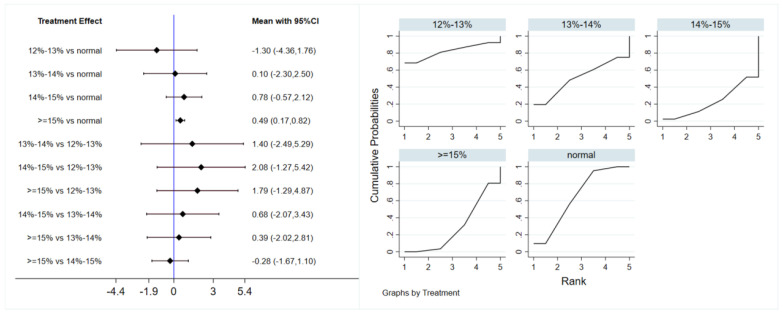
Network analysis of BMI with different oxygen volume fractions. The left panel displays a forest plot comparing BMI outcomes across different oxygen concentrations, while the right panel shows the SUCRA plot with the optimal intervention effect on BMI.

**Figure 3:**
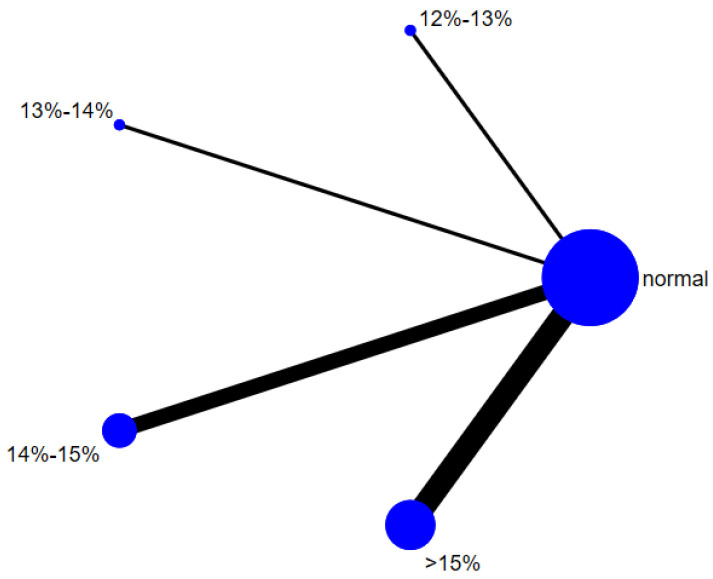
The reticular relationship of different oxygen volume fractions on BMI.

A total of 12 studies evaluated the effect of hypoxic exercise at different oxygen concentrations on the BMI of individuals with obesity, and a random-effects model was used for the network meta-analysis. The global inconsistency test yielded *p* = 0.311 (>0.05), indicating that there was no significant overall inconsistency among the included studies, and the consistency model was applicable. Figure 2 displays the pairwise comparison results of BMI reduction effects among different oxygen concentration groups, with the size of the squares representing the weight of each comparison, and the horizontal lines indicating the 95% CI. As shown in Table 2, While hypoxic exercise with ≥15% FiO_2_ showed a significant BMI reduction compared to normoxia (MD = −0.94, 95% CI: −1.82 to −0.17), the 12–14% FiO_2_ group demonstrated comprehensive benefits across multiple metabolic indicators (BMI, FBG, TG, LDL-C). Thus, the 12–14% range is recommended for overall metabolic improvement, while ≥15% FiO_2_ may be preferred for targeted BMI reduction. Figure 3 illustrates the network structure of direct and indirect comparisons between the four oxygen concentration groups and the normoxic group, showing that the ≥15% group had the most frequent direct comparisons with other groups (*n* = 4), followed by the 13–14% group (*n* = 3). Subgroup analysis revealed that, among exercise modalities, walking (MD = −0.50, 95% CI: −0.83 to −0.17, *p* < 0.05) had a more significant effect on BMI reduction than other forms of exercise. Regarding intervention duration, short-term programs (2–8 weeks) showed a significant effect (MD = −0.47, 95% CI: −0.79 to −0.14, *p* < 0.05). For frequency, interventions conducted 3–4 times per week were most effective (MD = −0.49, 95% CI: −0.81 to −0.17, *p* < 0.05), while session duration had no significant impact.

### 3.4. Indicators Related to Glucose Metabolism

#### 3.4.1. Fasting Blood Glucose

**Figure 4:**
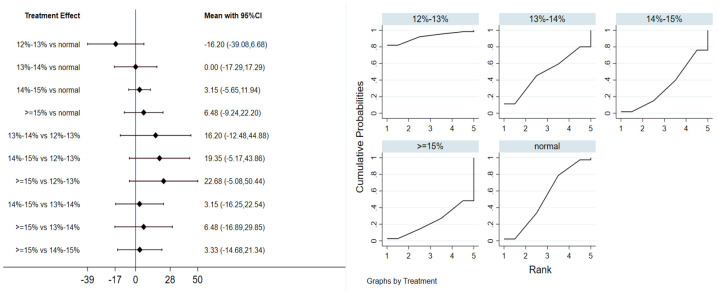
Network analysis of FBG with different oxygen volume fractions. The left panel displays a forest plot comparing FBG outcomes across different oxygen concentrations, while the right panel shows the SUCRA plot with the optimal intervention effect on FBG.

A total of 7 studies evaluated the effect of hypoxic exercise on FBG in individuals with obesity across different oxygen concentrations, and a meta-analysis was performed using a random-effects model. The global inconsistency test yielded *p* = 0.419 (>0.05), indicating that there was no significant overall inconsistency among the included studies, and the consistency model was applicable. Figure 4 presents the pooled effect sizes and 95% CI for FBG reduction in each oxygen concentration group compared to the normoxic group, with the rank plot on the right showing the probability of each group being the most effective. As shown in Table 3, hypoxic exercise at an oxygen concentration of 12% to 13% was more effective in reducing FBG than other concentrations. Subgroup analysis indicated that, for exercise modality, high-intensity interval training (MD = −7.87, 95% CI: −13.63 to −2.11, *p* < 0.05) had a more significant effect on FBG reduction than other forms of exercise. The total intervention period did not significantly influence the effect. Regarding frequency, interventions conducted more than 5 times per week were most effective (MD = −7.85, 95% CI: −13.57 to −2.13, *p* < 0.05). For session duration, exercise sessions longer than 60 min yielded the greatest benefit (MD = −8.76, 95% CI: −16.24 to −1.28, *p* < 0.05).

#### 3.4.2. Fasting Insulin

**Figure 5:**
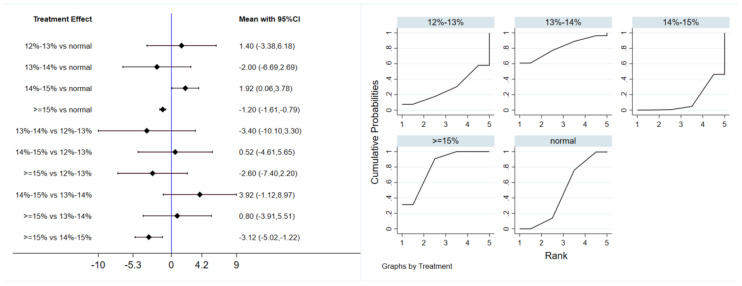
Network analysis of FINS with different oxygen volume fractions. The left panel displays a forest plot comparing FINS outcomes across different oxygen concentrations, while the right panel shows the SUCRA plot with the optimal intervention effect on FINS.

Six studies evaluated the effect of hypoxic exercise on FINS in individuals with obesity across different oxygen concentrations, using a random-effects model for the network meta-analysis. The global inconsistency test yielded *p* = 0.052 (>0.05), indicating that there was no significant overall inconsistency among the included studies, and the consistency model was applicable. The results, presented in Table 4 and Figure 5, indicate that hypoxic exercise at an oxygen concentration greater than 14% is more effective than normoxic exercise in reducing FINS. This effect was significant for the 14–15% range (MD = −1.92, 95% CI: −3.78 to −0.06) and for concentrations above 15% (MD = −1.20, 95% CI: −1.61 to −0.79). Subgroup analyses found that exercise modality, total intervention period, frequency, and session duration did not have significant effects on FINS.

#### 3.4.3. Homeostatic Model Assessment of Insulin Resistance

**Figure 6:**
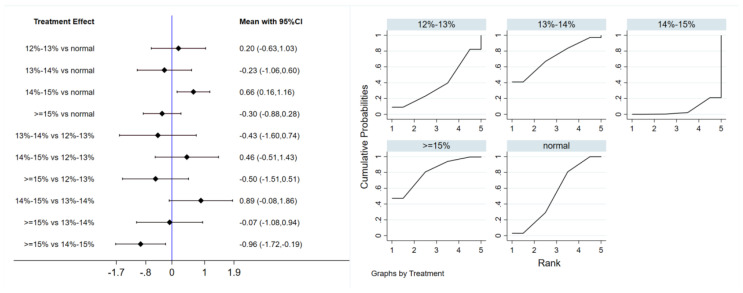
Network analysis of HOMA-IR with different oxygen volume fractions. The left panel displays a forest plot comparing HOMA-IR outcomes across different oxygen concentrations, while the right panel shows the SUCRA plot with the optimal intervention effect on HOMA-IR.

**Figure 7:**
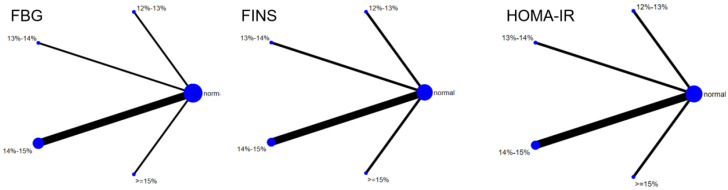
The reticular relationship of different oxygen volume fractions on glucose metabolism.

Six studies evaluated the effect of hypoxic exercise on the HOMA-IR in individuals with obesity across different oxygen concentrations, using a fixed-effects model for the network meta-analysis. The global inconsistency test yielded *p* = 0.307 (>0.05), indicating that there was no significant overall inconsistency among the included studies, and the consistency model was applicable. As shown in Table 5 and Figure 6, illustrates the pooled effect sizes of HOMA-IR reduction for each oxygen concentration group, with the forest plot-style structure showing the MD and 95% CI for each comparison, hypoxic exercise at an oxygen concentration of 14–15% was more effective than normoxic exercise in reducing HOMA-IR (MD = −0.66, 95% CI: −1.16 to −0.16). Furthermore, exercise conducted more than five times per week yielded the greatest benefit (MD = −0.90, 95% CI: −1.03 to −0.77, *p* < 0.001). For session duration, interventions lasting more than 60 min were most effective (MD = −0.87, 95% CI: −1.00 to −0.74, *p* < 0.001). Subgroup analyses found that exercise modality and total intervention period did not significantly influence HOMA-IR. Figure 7 summarizes the network comparison structure of the three glucose metabolism indicators (FBG, FINS, HOMA-IR) across different oxygen concentration groups, showing that the 14–15% FiO_2_ group had the most comprehensive positive effects on glucose metabolism.

### 3.5. Indicators Related to Lipid Metabolism

#### 3.5.1. Triglycerides

**Figure 8:**
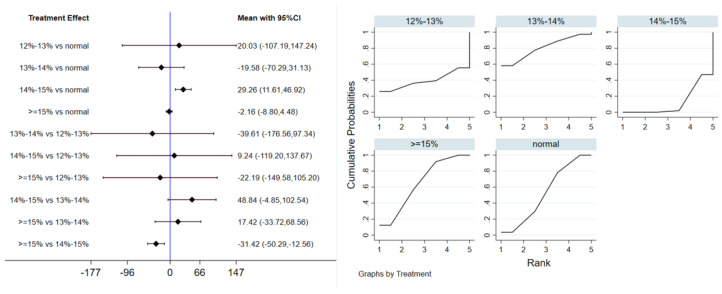
Network analysis of TG with different oxygen volume fractions. The left panel displays a forest plot comparing TG outcomes across different oxygen concentrations, while the right panel shows the SUCRA plot with the optimal intervention effect on TG.

Twelve studies evaluated the effect of hypoxic exercise on TG in individuals with obesity across different oxygen concentrations, using a random-effects model for meta-analysis. The global inconsistency test yielded *p* = 0.524 (>0.05), indicating that there was no significant overall inconsistency among the included studies, and the consistency model was applicable. As shown in Table 6, Figure 8 presents the pairwise comparison results of TG reduction, with the size of the circles representing the sample size of each group, and the connecting lines indicating direct comparisons, hypoxic exercise was more effective than normoxic exercise in reducing TG, with the most significant effects observed at oxygen concentrations of 13% to 14% and 14% to 15% (e.g., 14–15%: MD = −29.26, 95% CI: −46.92 to −11.61). Subgroup analysis revealed that, regarding intervention duration, programs lasting 12–24 weeks were most effective (MD = −29.49, 95% CI: −56.02 to −2.96, *p* < 0.05). For frequency, interventions conducted 1–2 times per week showed the greatest benefit (MD = −32.74, 95% CI: −59.17 to −6.31, *p* < 0.05), while session duration had no significant impact.

#### 3.5.2. Low-Density Lipoprotein Cholesterol

**Figure 9:**
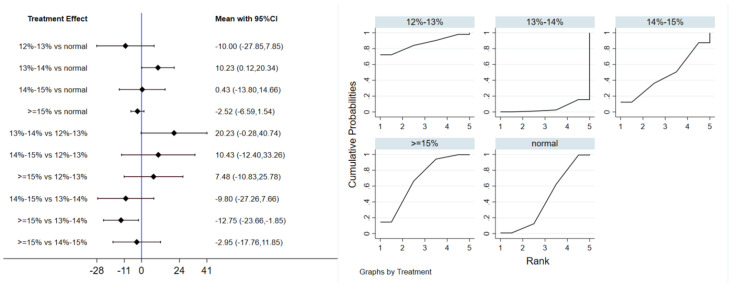
Network analysis of LDL-C with different oxygen volume fractions. The left panel displays a forest plot comparing LDL-C outcomes across different oxygen concentrations, while the right panel shows the SUCRA plot with the optimal intervention effect on LDL-C.

Eleven studies evaluated the effect of hypoxic exercise on LDL-C in individuals with obesity across different oxygen concentrations, using a fixed-effects model for meta-analysis. The global inconsistency test yielded *p* = 0.224 (>0.05), indicating that there was no significant overall inconsistency among the included studies, and the consistency model was applicable. As shown in Table 7, Figure 9 displays the pooled effect sizes of LDL-C reduction, with the rank order on the right indicating the relative effectiveness of each oxygen concentration group, hypoxic exercise with 12–14% FiO_2_ showed a modest but significant reduction in LDL-C (MD = −10.23, 95% CI: −20.34 to −0.12), with the absolute reduction approaching the clinical relevance threshold. This suggests potential clinical value for long-term dyslipidemia management. Subgroup analysis indicated that, among exercise modalities, Pilates showed a more pronounced effect on LDL-C reduction than other forms of exercise (MD = −37.10, 95% CI: −73.80 to −0.40, *p* = 0.05). In contrast, the total intervention period, weekly frequency, and session duration did not demonstrate a significant impact on LDL-C outcomes.

#### 3.5.3. High-Density Lipoprotein Cholesterol

**Figure 10:**
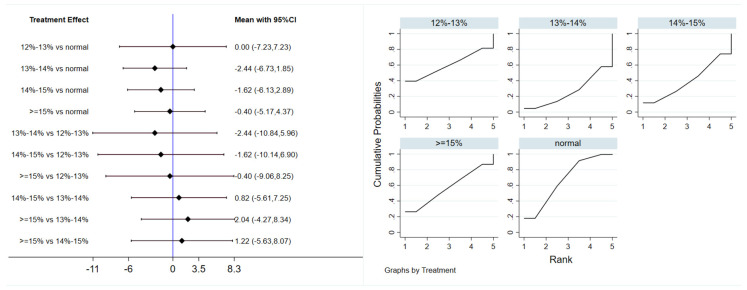
Network analysis of HDL-C with different oxygen volume fractions. The left panel displays a forest plot comparing HDL-C outcomes across different oxygen concentrations, while the right panel shows the SUCRA plot with the optimal intervention effect on HDL-C.

**Figure 11:**
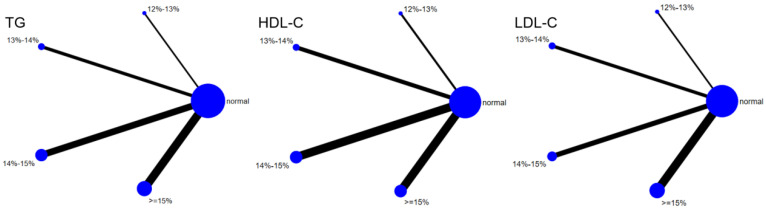
The reticular relationship of different oxygen volume fractions on lipid metabolism.

Eleven studies evaluated the effect of hypoxic exercise on HDL-C in individuals with obesity across different oxygen concentrations, using a random-effects model for network meta-analysis. The global inconsistency test yielded *p* = 0.868 (>0.05), indicating that there was no significant overall inconsistency among the included studies, and the consistency model was applicable. As shown in Table 8, Figure 10 illustrates the pairwise comparison results of HDL-C changes, with the horizontal lines indicating non-significant 95% CI for all comparisons, consistent with the overall non-significant effect, the overall analysis did not show a statistically significant effect of hypoxic exercise on HDL-C. However, the SUCRA analysis suggested that exercise in a normoxic environment may be more effective for improving HDL-C than hypoxic conditions. Subgroup analyses further indicated that exercise modality, total intervention period, frequency, and session duration had no significant impact on HDL-C. Figure 11 summarizes the network comparison structure of the three lipid metabolism indicators (TG, HDL-C, LDL-C) across different oxygen concentration groups, showing that the 13–14% and 14–15% FiO_2_ groups had concentrated positive effects on TG and LDL-C reduction.

### 3.6. Risk of Bias and Methodological Quality

**Figure 12:**
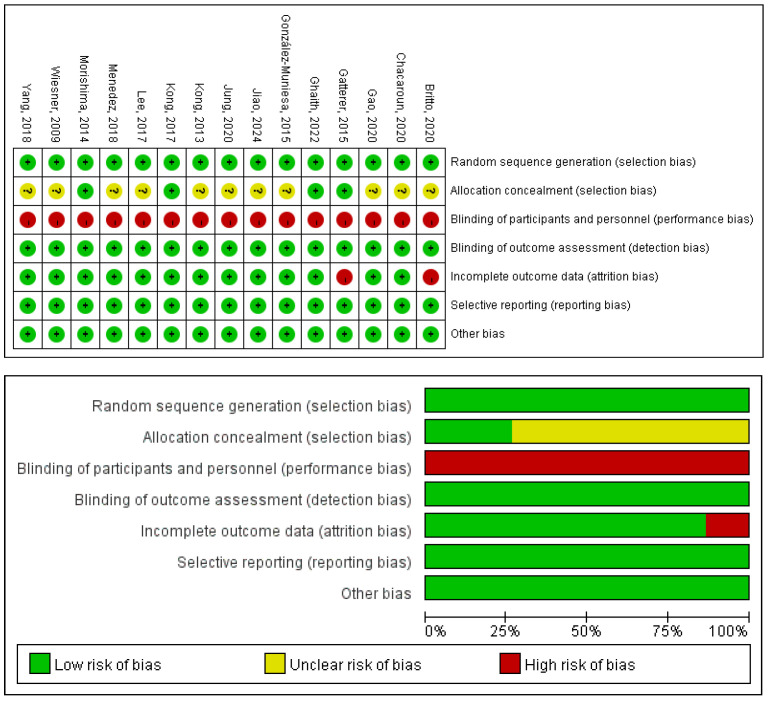
Risk of bias of the included studies [25,26,27,28,29,30,31,32,33,34,35,36,37,38,41].

The quality of the 14 included RCTs was assessed using the Cochrane Risk of Bias tool [25,26,27,28,29,30,31,32,33,34,35,36,37,38], and Review Manager 5.4. Detailed results of the literature quality evaluation are presented in Figure 12, the evaluation focused on the following aspects: (1) Selection bias: whether the method of random sequence generation was used; (2) Allocation concealment: whether the allocation was effectively concealed; (3) Blinding: whether the subjects or investigators were blinded; (4) Data integrity: whether missing data was adequately reported and intention-to-treat analysis was applied; (5) Selective reporting: whether there was any selective reporting of outcomes; (6) Other biases: whether other factors contributed to potential bias.

The methodological quality of the included studies was appraised using the PEDro scale; the detailed ratings are presented in Table 9. The mean PEDro score across all studies was 6.07, reflecting an overall good level of methodological rigor. According to conventional interpretation of the scale, the studies were categorized as follows: 64% were of good quality (scores 6–8), 29% were of fair quality (scores 4–5), and 7% were of poor quality (scores ≤3).

### 3.7. Certainty of the Evidence 

Overall, the certainty of evidence ranged from very low to moderate across the assessed outcomes, as shown in Table 10. Moderate certainty was found for the effects on Fasting insulin and Homeostatic Model Assessment of Insulin Resistance. In contrast, most other outcomes, including Body mass index, Fasting blood glucose, Triglycerides, Low-density lipoprotein cholesterol and High-density lipoprotein cholesterol, were supported by evidence of low to very low certainty.

Overall, the certainty of evidence for the assessed outcomes ranged from very low to moderate, as summarized in Table 3. The certainty was rated as moderate for fasting insulin and the HOMA-IR. For all other outcomes—including BMI, FBG, TG, LDL-C, and HDL-C—the certainty of evidence was low to very low.

### 3.8. Sensitivity Analysis

To evaluate the robustness of the meta-analytic results, a leave-one-out sensitivity analysis was performed for outcomes exhibiting substantial heterogeneity. The analysis revealed that neither the direction nor the statistical significance of the pooled effect estimate was substantially altered by the sequential exclusion of any single study, confirming the reliability of the findings. Furthermore, while the exclusion of individual studies occasionally lowered the I^2^ statistic, the overall impact on heterogeneity estimation was minimal. This confirms that no single study exerted a disproportionate influence on the results. 

### 3.9. Heterogeneity Analysis

Heterogeneity was assessed through pairwise comparisons, Cochran’s Q test, and global and local inconsistency tests for each outcome. The global test indicated no significant overall heterogeneity across the included metabolic indicators (*p* > 0.05). Cochran’s Q test revealed significant heterogeneity for FBG, FINS, and HOMA-IR (*p* < 0.05, I^2^ > 50%), while other indicators were homogeneous. Further analysis identified specific sources of heterogeneity. Both BMI and FINS were influenced by the type of intervention; BMI heterogeneity originated primarily from AT and RT, whereas fasting insulin heterogeneity was linked to treadmill training. This suggests that different metabolic indicators vary in their sensitivity to intervention modality. For FBG, the core source of heterogeneity was intervention duration, indicating that acute glycemic regulation is closely tied to the length of the exercise session. Furthermore, heterogeneity in BMI and FBG was also driven by intervention frequency: BMI by 1–2 times/week and fasting blood glucose by 2–4 times/week, reflecting distinct response thresholds to frequency across metabolic outcomes. Finally, FINS and FBG shared a common source of heterogeneity in the intervention cycle (2–8 weeks). This implies that short-term interventions produce unstable effect sizes on the glucose-insulin axis, likely due to the high sensitivity of these metabolic markers to short-term fluctuations.

## 4. Discussion

This study systematically evaluates the effects of hypoxic exercise at different oxygen concentrations on metabolic health in individuals with obesity. The results demonstrate that hypoxic exercise can induce significant improvements in BMI, glucose metabolism, and specific lipid parameters. Based on the study results, we propose the following practical guidelines for hypoxic exercise interventions in obese populations. For comprehensive metabolic improvement (BMI, FBG, TG, LDL-C), consider 12–14% FiO_2_ with 3–4 times/week of aerobic + resistance training (≥60 min/session, 2–8 weeks). For insulin sensitivity (FINS, HOMA-IR), ≥15% FiO_2_ with ≥5 times/week (≥60 min/session) is suggested. These recommendations are preliminary and should be implemented under professional monitoring, with individualization based on baseline health status. Avoid extremely low oxygen concentration (<12% FiO_2_): Severe hypoxia may trigger stress responses, impair insulin signaling, and increase metabolic burden [40], leading to no significant improvement in glucose metabolism indicators.

Although the precise mechanisms through which hypoxia influences metabolism are not fully understood, the prevailing hypothesis suggests that a hypoxic environment confers metabolic benefits by activating the HIF-1α signaling pathway [23,41]. This activation is proposed to enhance fat oxidation, suppress lipogenesis, and improve insulin sensitivity. Hypoxic exercise demonstrates a positive impact on glucose metabolism and insulin resistance in individuals with obesity. The findings of this study indicate significant reductions in both FBG and FINS, with the most pronounced improvements observed in a moderate hypoxia environment of 13% oxygen concentration. Hypoxic exercise with 14–15% FiO_2_ was more effective than normoxia in reducing HOMA-IR. This finding aligns with the results of a controlled trial by Mai et al. [42], which reported that hypoxic exercise enhanced insulin sensitivity and glucose regulation in participants. The metabolic advantages of hypoxia are supported by multiple lines of evidence. Populations residing long-term at high altitudes exhibit a lower incidence of diabetes compared to those in plains. Hamlin et al. [43], found that short-term exercise at high altitude significantly reduced insulin resistance and postprandial blood glucose concentration. Furthermore, Zhao et al. [44], using a diabetic mouse model, demonstrated that short-term moderate hypoxia exposure enhanced mitochondrial NAD cycle activity, suggesting a mechanism by which hypoxia may ameliorate glucose metabolism disorders through improved mitochondrial function. These studies collectively confirm that both chronic and acute hypoxia exposure can beneficially regulate glucose metabolism, although the activation pathways may differ between continuous hypoxia and intermittent hypoxic exercise. Presently, a systematic comparison clarifying their respective advantages and optimal target populations is lacking. The underlying mechanisms involve enhanced glucose uptake in skeletal muscle, primarily regulated through the expression and translocation of GLUT-4 to the cell membrane via multiple signaling pathways [45]. This process enhances glucose utilization, thereby improving insulin sensitivity and maintaining homeostasis. Additionally, hypoxic exercise can inhibit the release of inflammatory factors such as TNF-α and IL-6, mitigating chronic low-grade inflammation’s interference with insulin signaling and further consolidating its metabolic benefits [46]. However, this study found that an extremely low oxygen environment (FiO_2_ < 12%) did not significantly improve glucose metabolism. This is consistent with Chacaroun et al. [32], who observed no significant improvement in glucose metrics with a lower FiO_2_ in a short-term pilot study. This lack of efficacy may be attributed to a stress response induced by severe hypoxia, which can impair insulin signaling and increase metabolic burden [40]. Therefore, the therapeutic window for hypoxic exercise requires careful selection of an oxygen concentration that promotes metabolic adaptation without triggering a counterproductive stress response.

Furthermore, hypoxic exercise demonstrates a significant beneficial impact on lipid metabolism in individuals with obesity, characterized by pronounced reductions in TG and LDL-C, whereas HDL-C levels show no significant improvement. This lipid-lowering effect is most prominent in a moderate hypoxic environment with an oxygen concentration below 14%. These findings are consistent with prior research, reinforcing the superiority of hypoxic over normoxic exercise for reducing TG and LDL-C. For instance, Morishima et al. [41] reported that aerobic training in hypoxia effectively promotes lipid metabolism, specifically improving visceral fat and dyslipidemia in sedentary obese women. The efficacy of lipid metabolism improvement is moderated by several factors, with exercise modality and intensity being particularly critical. Both HIIT and moderate-intensity continuous aerobic exercise have demonstrated robust lipid-regulating effects under hypoxic conditions. However, the influence of modality is evident in the study by Jung et al. [34], which utilized Pilates at 14.5% FiO_2_ and observed no significant changes in lipid profiles. Regarding HDL-C, existing evidence presents considerable heterogeneity. Many studies indicate that short-term hypoxic intervention is insufficient to elicit a significant increase in HDL-C. This is likely attributable to the complex synthesis pathway of HDL-C, which may depend more on prolonged, moderate to high intensity exercise and a systematic amelioration of the overall lipid metabolic environment to be effectively upregulated. Supporting this, Brito et al. [25], documented a mild increase in HDL-C following a 30-week hypoxic training program in adolescents, suggesting that extended intervention duration is a key factor for an HDL-C response [40]. Individual differences also significantly modulate lipid outcomes. Evidence suggests that post-intervention increases in HDL-C are generally more pronounced in women than in men, potentially due to estrogen’s regulatory role in lipid metabolism [47]. Concurrently, individuals with elevated baseline lipid levels or metabolic abnormalities tend to experience more substantial reductions in TG and LDL-C, while changes in HDL-C remain limited, particularly following short-term or low-intensity interventions.

## 5. Limitations

This study has several limitations that highlight important avenues for future research. First, the sample size was limited, with only 189 participants across all included studies. Several subgroup analyses also contained few studies, which may compromise the stability of the findings. Future multi-center randomized controlled trials with larger sample sizes are needed to verify the optimal hypoxic oxygen concentration. Second, there was notable heterogeneity in intervention parameters—particularly in cycle length and exercise intensity—across the included studies. And the limited use of normoxic control-group data in the primary network meta-analysis, which may restrict the ability to quantify the absolute effect of hypoxic exercise compared to conventional normoxic exercise. Standardizing these parameters in future work would reduce heterogeneity and strengthen the reliability of the results. Third, we did not examine differential intervention effects by gender, age, or baseline metabolic status. Stratified analyses in subsequent studies could help determine the optimal hypoxic exercise prescription for specific sub-groups. Finally, most interventions lasted less than one year, so the long-term maintenance of metabolic benefits and the cardiovascular safety of sustained hypoxic training remain unclear. Long-term follow-up studies are therefore required to evaluate the sustainability and safety of this intervention.

## 6. Conclusions

This systematic review and network meta-analysis provides preliminary evidence that hypoxic exercise can improve BMI, glucose metabolism, and selected lipid parameters in obese individuals. Moderate hypoxia (12–14% FiO_2_) may benefit BMI, FBG, TG, and LDL-C, while higher concentrations (≥15% FiO_2_) may improve FINS and HOMA-IR. As a key parameter, the precise calibration of oxygen concentration is crucial for optimizing intervention efficacy. Consequently, tailored exercise in specific hypoxic environments presents a promising non-pharmacological intervention strategy for the health management of obesity.

## Figures and Tables

**Table 1:** Characteristics of the studies included.

Author/Year	N	BMI	Age	Oxygen Volume Fraction	Types	Time	Frequency	Period	Indicators
Britto, 2020 [25]	7	35.4 ± 4.2	12–17	15%	AT + RT	50–60 min	1 times/week	30 weeks	BMI
González-Muniesa, 2015 [26]	17	34.4 ± 2.8	25–50	16–13.7%	AT + RT	60 min	2 times/week	8 weeks	BMI, TG, HDL-C, LDL-C
Gatterer, 2015 [27]	16	37.9 ± 8.1	50.3 ± 10.3	14%	AT	90 min	2 times/week	32 weeks	BMI, FBG, TG, HDL-C
Gao, 2020 [28]	10	28 ± 3.6	19.30 ± 1.92	15.90%	AT	120 min	3 times/week	4 weeks	TG, HDL-C, LDL-C
Lee, 2017 [29]	10	26.3 ± 4.0	38 ± 8.2	13.50%	AT	60 min	3 times/week	12 weeks	TG, HDL, LDL-C
Kong, 2017 [30]	14	25.8 ± 2.3	18–30	15.00%	HIIT	20 min	4 times/week	5 weeks	BMI, TG, HDL-C, LDL-C
Yang, 2018 [31]	16	32.9 ± 3.5	14.3 ± 1.4	14.70%	AT	120 min	6 times/week	4 weeks	BMI, FBG, FI, HOMA-IR, TG, HDL-C, LDL-C
Chacaroun, 2020 [32]	12	31.2 ± 2.4	52 ± 12	13%	AT	45 min	3 times/week	8 weeks	BMI, FBG, FI, HOMA-IR, TG, HDL-C, LDL-C
Kong, 2013 [33]	10	34.7 ± 5.3	19.8 ± 2.2	16.4–14.5%	AT + RT	120 min	11 times/week	4 weeks	BMI
Jung, 2020 [34]	12	27.1 ± 4.3	47.2 ± 6.4	14.50%	AT	50 min	3 times/week	12 weeks	BMI, FBG, FI, HOMA-IR, TG, HDL-C, LDL-C
Jiao, 2024 [35]	13	28.1 ± 2.1	36.62 ± 9.54	15%	AT + RT	60 min	5 times/week	4 weeks	BMI, FBG, TG, HDL-C, LDL-C
Menedez, 2018 [36]	12	34.1 ± 2.6	34.8 ± 4.7	14.50%	AT	60 min	3 times/week	3 weeks	BMI, FBG, FI, HOMA-IR, TG, HDL-C, LDL-C
Ghaith, 2022 [37]	16	31.5 ± 4	51.0 ± 8.3	12%	HIIT	50 min	3 times/week	8 weeks	BMI, FBG, FI, HOMA-IR, TG, HDL-C, LDL-C
Wiesner, 2009 [38]	24	30.2 ± 3.6	42.2 ± 1.2	15%	AT	60 min	3 times/week	4 weeks	BMI, FI, HOMA-IR, TG, LDL-C

**Table 2:** League table of the influence of different oxygen volume fractions on BMI.

**Normal**	0.49 (0.17,0.82)	0.78 (−0.57,2.12)	0.10 (−2.30,2.50)	−1.30 (−4.36,1.76)
−0.49 (−0.82,−0.17)	**≥15%**	0.28 (−1.10,1.67)	−0.39 (−2.81,2.02)	−1.79 (−4.87,1.29)
−0.78 (−2.12,0.57)	−0.28 (−1.67,1.10)	**14–15%**	−0.68 (−3.43,2.07)	−2.08 (−5.42,1.27)
−0.10 (−2.50,2.30)	0.39 (−2.02,2.81)	0.68 (−2.07,3.43)	**13–14%**	−1.40 (−5.29,2.49)
1.30 (−1.76,4.36)	1.79 (−1.29,4.87)	2.08 (−1.27,5.42)	1.40 (−2.49,5.29)	**12–13%**

**Table 3:** League table of the influence of different oxygen volume fractions on FBG.

**Normal**	−16.20 (−39.08,6.68)	0.00 (−17.29,17.29)	3.15 (−5.65,11.94)	6.48 (−9.24,22.20)
16.20 (−6.68,39.08)	**12–13%**	16.20 (−12.48,44.88)	19.35 (−5.17,43.86)	22.68 (−5.08,50.44)
−0.00 (−17.29,17.29)	−16.20 (−44.88,12.48)	**13–14%**	3.15 (−16.25,22.54)	6.48 (−16.89,29.85)
−3.15 (−11.94,5.65)	−19.35 (−43.86,5.17)	−3.15 (−22.54,16.25)	**14–15%**	3.33 (−14.68,21.34)
−6.48 (−22.20,9.24)	−22.68 (−50.44,5.08)	−6.48 (−29.85,16.89)	−3.33 (−21.34,14.68)	**≥15%**

**Table 4:** League table of the influence of different oxygen volume fractions on FINS.

**Normal**	1.40 (−3.38,6.18)	−2.00 (−6.69,2.69)	1.92 (0.06,3.78)	−1.20 (−1.61,−0.79)
−1.40 (−6.18,3.38)	**12–13%**	−3.40 (−10.10,3.30)	0.52 (−4.61,5.65)	−2.60 (−7.40,2.20)
2.00 (−2.69,6.69)	3.40 (−3.30,10.10)	**13–14%**	3.92 (−1.12,8.97)	0.80 (−3.91,5.51)
−1.92 (−3.78,−0.06)	−0.52 (−5.65,4.61)	−3.92 (−8.97,1.12)	**14–15%**	−3.12 (−5.02,−1.22)
1.20 (0.79,1.61)	2.60 (−2.20,7.40)	−0.80 (−5.51,3.91)	3.12 (1.22,5.02)	**≥15%**

**Table 5:** League table of the influence of different oxygen volume fractions on HOMA-IR.

**Normal**	0.20 (−0.63,1.03)	−0.23 (−1.06,0.60)	0.66 (0.16,1.16)	−0.30 (−0.88,0.28)
−0.20 (−1.03,0.63)	**12–13%**	−0.43 (−1.60,0.74)	0.46 (−0.51,1.43)	−0.50 (−1.51,0.51)
0.23 (−0.60,1.06)	0.43 (−0.74,1.60)	**13–14%**	0.89 (−0.08,1.86)	−0.07 (−1.08,0.94)
−0.66 (−1.16,−0.16)	−0.46 (−1.43,0.51)	−0.89 (−1.86,0.08)	**14–15%**	−0.96 (−1.72,−0.19)
0.30 (−0.28,0.88)	0.50 (−0.51,1.51)	0.07 (−0.94,1.08)	0.96 (0.19,1.72)	**≥15%**

**Table 6:** League table of the influence of different oxygen volume fractions on TG.

**Normal**	20.03 (−107.19,147.24)	−19.58 (−70.29,31.13)	29.26 (11.61,46.92)	−2.16 (−8.80,4.48)
−20.03 (−147.24,107.19)	**12–13%**	−39.61 (−176.56,97.34)	9.24 (−119.20,137.67)	−22.19 (−149.58,105.20)
19.58 (−31.13,70.29)	39.61 (−97.34,176.56)	**13–14%**	48.84 (−4.85,102.54)	17.42 (−33.72,68.56)
−29.26 (−46.92,−11.61)	−9.24 (−137.67,119.20)	−48.84 (−102.54,4.85)	**14–15%**	−31.42 (−50.29,−12.56)
2.16 (−4.48,8.80)	22.19 (−105.20,149.58)	−17.42 (−68.56,33.72)	31.42 (12.56,50.29)	**≥15%**

**Table 7:** League table of the influence of different oxygen volume fractions on LDL-C.

**Normal**	−10.00 (−27.85,7.85)	10.23 (0.12,20.34)	0.43 (−13.80,14.66)	−2.52 (−6.59,1.54)
10.00 (−7.85,27.85)	**12–13%**	20.23 (−0.28,40.74)	10.43 (−12.40,33.26)	7.48 (−10.83,25.78)
−10.23 (−20.34,−0.12)	−20.23 (−40.74,0.28)	**13–14%**	−9.80 (−27.26,7.66)	−12.75 (−23.66,−1.85)
−0.43 (−14.66,13.80)	−10.43 (−33.26,12.40)	9.80 (−7.66,27.26)	**14–15%**	−2.95 (−17.76,11.85)
2.52 (−1.54,6.59)	−7.48 (−25.78,10.83)	12.75 (1.85,23.66)	2.95 (−11.85,17.76)	**≥** **15%**

**Table 8:** League table of the influence of different oxygen volume fractions on HDL-C.

**Normal**	−0.00 (−7.23,7.23)	−2.44 (−6.73,1.85)	−1.62 (−6.13,2.89)	−0.40 (−5.17,4.37)
0.00 (−7.23,7.23)	**12–13%**	−2.44 (−10.84,5.96)	−1.62 (−10.14,6.90)	−0.40 (−9.06,8.25)
2.44 (−1.85,6.73)	2.44 (−5.96,10.84)	**13–14%**	0.82 (−5.61,7.25)	2.04 (−4.27,8.34)
1.62 (−2.89,6.13)	1.62 (−6.90,10.14)	−0.82 (−7.25,5.61)	**14–15%**	1.22 (−5.63,8.07)
0.40 (−4.37,5.17)	0.40 (−8.25,9.06)	−2.04 (−8.34,4.27)	−1.22 (−8.07,5.63)	**≥15%**

**Table 9:** PEDro scale assessment results.

Author, Year	D1	D2	D3	D4	D5	D6	D7	D8	D9	D10	D11	Total
Britto, 2020 [25]	Y	1	1	1	0	0	**1**	1	1	1	1	8
González-Muniesa, 2015 [26]	Y	1	1	1	0	0	1	1	1	1	1	8
Gatterer, 2015 [27]	Y	1	0	1	0	0	1	1	1	1	1	7
Gao, 2020 [28]	Y	1	1	1	0	0	1	1	1	1	1	8
Lee, 2017 [29]	Y	1	0	0	0	0	0	1	1	1	1	5
Kong, 2017 [30]	Y	1	0	0	0	0	0	0	1	1	1	4
Yang, 2018 [31]	Y	1	1	1	0	0	1	1	1	1	1	8
Chacaroun, 2020 [32]	N	1	0	0	0	0	0	1	1	1	1	5
Kong, 2013 [33]	Y	1	0	1	0	0	0	1	1	1	1	6
Jung, 2020 [34]	Y	1	1	1	0	0	0	1	1	1	1	7
Jiao, 2024 [35]	Y	0	0	0	0	0	0	1	1	1	1	4
Menedez, 2018 [36]	Y	1	1	1	0	0	1	0	1	1	1	7
Ghaith, 2022 [37]	Y	1	1	0	0	0	1	0	1	1	1	6
Wiesner, 2009 [38]	Y	0	0	0	0	0	0	0	1	0	1	2

Note: studies scoring ≥ 6 are considered high quality, those scoring 4–5 are considered moderate quality, and those scoring ≤ 3 are considered low quality. 1. Eligibility criteria were specified (not included in the total score). 2. The subjects were randomly allocated to groups (in a crossover study, the subjects were randomly allocated in an order in which the treatments were received). 3. Allocation was concealed. 4. The groups were similar at baseline in terms of the most important prognostic indicators. 5. All the subjects were blinded. 6. All the therapists who administered the therapy were blinded. 7. All assessors who measured at least one key outcome were blinded. 8. Measures of at least one key outcome were obtained from more than 85% of the subjects initially allocated to groups. 9. All subjects for whom outcome measures were available received the treatment or control condition as allocated or, where this was not the case, data for at least one key outcome were analyzed by “intention to treat”. 10. the results of between-group statistical comparisons are reported for at least one key outcome. 11. the study provides both point measures and measures of variability for at least one key outcome.

**Table 10:** GRADE.

Outcome	No of Participants (Studies)	Certainty Assessment					Standardized Mean Effect (95% CI) †	Grade *
		Risk of Bias	Inconsistency	Indirectness	Imprecision	Other		
Body Composition								
BMI	12 RCTs	Very serious	Serious	Serious	Not serious	None	−2.29 [−3.42,−1.17]	⨁⨁◯◯ Low
Glucose metabolism								
FBG	7 RCTs	Very serious	Serious	Serious	Not serious	None	−3.58 [−6.32,−0.93]	⨁⨁◯◯ Low
FINS	6 RCTs	Very serious	Not serious	Serious	Not serious	None	−1.60 [−2.98,0.22]	⨁⨁⨁◯ Moderate
HOMA-IR	6 RCTs	Very serious	Not serious	Serious	Not serious	None	−0.74 [−1.52,0.04]	⨁⨁⨁◯ Moderate
lipid metabolism								
TG	12 RCTs	Very serious	Serious	Serious	Not serious	None	−0.18 [−0.25,−0.12]	⨁⨁◯◯ Low
LDL-C	11 RCTs	Very serious	Serious	Serious	Not serious	None	−0.25 [−0.9,−0.11]	⨁⨁◯◯ Low
HDL-C	11 RCTs	Very serious	Not serious	Serious	Serious	None	−0.09 [−0.21,0.02]	⨁⨁◯◯ Low

* Certainty of evidence according to Grading of Recommendations, Assessment, Development and Evaluations (GRADE): High: We are very confident in the estimated effect. Moderate: Our confidence in the estimated effect is moderate. Low: We have limited confidence in the estimated effect. Very low: We have very little confidence in the estimated effect. †: Refer to the following notes to explain the details of the evidence, ⨁: Representative of valid/sufficient evidence units, ◯: Representative of weak/insufficient evidence units.

## Data Availability

The corresponding author of this article will unconditionally provide all the original data supporting the results of this study.

## Supplementary Materials

The following supporting information can be downloaded at: https://www.mdpi.com/article/10.3390/life16020231/s1, Table S1. PRISMA.
